# Finding common ground: meta-synthesis of communication frameworks found in patient communication, supervision and simulation literature

**DOI:** 10.1186/s12909-019-1922-2

**Published:** 2020-02-11

**Authors:** Matthew Jon Links, Leonie Watterson, Peter Martin, Stephanie O’Regan, Elizabeth Molloy

**Affiliations:** 1 Gold Coast University Hospital and Health Service, Southport, Australia; 2 Griffith University Institute of Educational Research and School of Medicine, Brisbane, Australia; 3 Medical Oncology, 1 Hospital Boulevarde, Southport, QLD 4215 Australia; 4 Sydney Clinical Skills and Simulation Centre, Royal North Shore Hospital Sydney, Sydney, New South Wales Australia; 5 Deakin University Faculty of Health, School of Medicine, Geelong, Australia; 6 Department of Medical Education, University of Melbourne, Melbourne, Australia

**Keywords:** Communication, Skills, Education, Training, Supervision, Simulation, Power, Patient care

## Abstract

**Background:**

Effective communication between patients-clinicians, supervisors-learners and facilitators-participants within a simulation is a key priority in health profession education. There is a plethora of frameworks and recommendations to guide communication in each of these contexts, and they represent separate discourses with separate communities of practice and literature. Finding common ground within these frameworks has the potential to minimise cognitive load and maximise efficiency, which presents an opportunity to consolidate messages, strategies and skills throughout a communication curriculum and the possibility of expanding the research agenda regarding communication, feedback and debriefing in productive ways.

**Methods:**

A meta-synthesis of the feedback, debriefing and clinical communication literature was conducted to achieve these objectives.

**Results:**

Our analysis revealed that the concepts underlying the framework can be usefully categorised as stages, goals, strategies, micro-skills and meta-skills. Guidelines for conversations typically shared a common structure, and strategies aligned with a stage. Core transferrable communication skills (i.e., micro-skills) were identified across various types of conversation, and the major differences between frameworks were related to the way that power was distributed in the conversation and the evolution of conversations along the along the path of redistributing power. As part of the synthesis, an overarching framework “prepare-EMPOWER enact” was developed to capture these shared principles across discourses**.**

**Conclusions:**

Adopting frameworks for work-based communication that promote dialogue and empower individuals to contribute may represent an important step towards learner-centred education and person-centred care for patients.

## Background

Conversations are at the heart of patient care and education. A number of recent studies have indicated that clinical communication skills have an impact on patient outcomes [1–3]. In clinical and educational practices, multiple patient-practitioner, learner-supervisor and team-based conversations are employed to learn, adapt and co-construct. Eventually, the learner may become a supervisor who teaches communication skills and provides feedback and/or debriefing conversations relevant to this role. Teaching conversational approaches are therefore central to clinical education throughout a lifetime of learning designed to prepare learners for their various roles as clinicians, colleagues, supervisors, educators and learners [4].

Educators face significant challenges when teaching communication in these different contexts, even though many of the skills, strategies and overarching values are fairly common. There are different discourses, communities of practice, journals for publication and often different underpinning theoretical traditions; all of which are grounded in historical precedent. Consequently, the existing literature has a major shortcoming because knowledge is largely limited to its particular context, and few attempts have been made to achieve consistency in the quality of approaches across other contexts or to translate knowledge from one person to another [4].

Communication is a multifaceted construct that involves the appropriate application of certain core transferable skills and strategies, which is determined by the purpose of the communication, the participants, and the context. Some communication approaches are specific to particular contexts (such as simulation), which other conversations may be enacted across situations with the help of values that guide practice (e.g., the communication of “bad news” to a patient or peer). Mastering communication requires the consolidation of core skills and the ability to apply context-specific skills and strategies. Medical schools and colleges have responded to these challenges by embedding communication education into integrated curricula [5, 6] One model for an integrated communication curriculum is referred to as a spiral curriculum [7], and as learners come to communication tasks, they can revisit and reinforce knowledge obtained from previous tasks in a helical learning pattern. Such a model is enhanced by a consistent approach to communication education, though this has been difficult to achieve thus far, which is primarily due to substantial heterogeneity and limitations associated with published research. We believe that examining approaches’ shared properties across contexts provides an opportunity to teach common messages and reinforce core values and strategies as learners move through their lifelong education.

### Aim

Our research aim was to challenge the existing siloed communication teaching approaches across clinical and education conversations presented in the literature by identifying underlying structural elements and recommendations that are common to conversations between patients and clinicians in the clinical literature and between supervisors and learners in the educational literature. Shared properties could be synthesised into a unified structure with recommendations to guide the conduct and teaching of these conversations across this range of contexts, which may affect the teaching of communication in health profession programs and the research agenda associated with person-centred communication approaches in the healthcare field. A common framework may help build bridges between different but overlapping communities of practice that encompass patient communication, simulation and supervision research. A common framework would be useful to communication curricula designers and those who teach and research such skills.

## Methods

Identifying an appropriate method to address this question is problematical. A communication framework is a combination of empirical observation, a theoretical construct and the basis of an educational intervention. An appropriate method was developed based previous synthesis efforts, though it challenges assumptions and learns from alternative constructions and literature. A variety of methods were considered, but a mixed method based upon meta-synthesis was employed. Meta-synthesis is an interpretive integration of qualitative findings in primary studies that take the form of interpretive data synthesis, employing either conceptual/thematic descriptions or interpretive explanations.

**Fig. 1:**
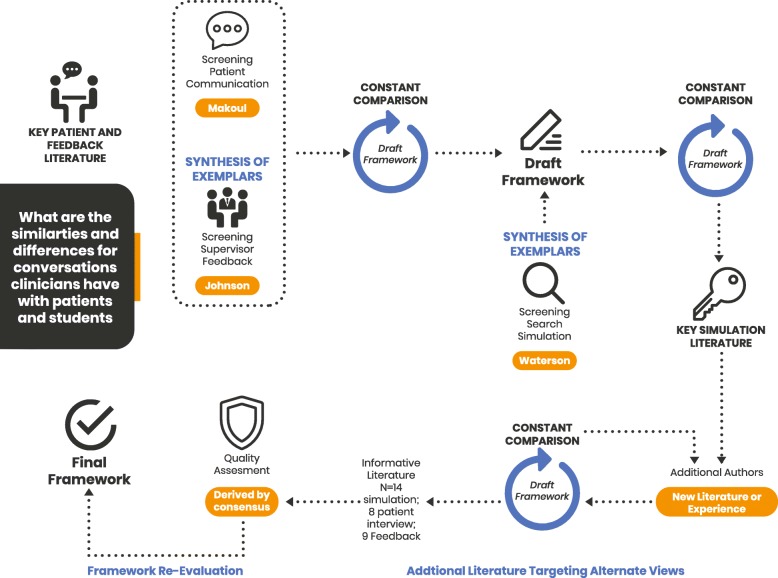
Meta-Synthesis methods. In response to the study question an iterative process of screening (literature search) synthesis into a draft framework and re-evaluation was undertaken. An exemplar was chosen from each of the three conversations studied (Makoul, Johnson and Watterson) to initiate the process. A process of constant comparison between an identified study and the draft framework was used to challenge and improve the existing framework

We therefore conducted a meta-synthesis of published frameworks designed to structure patient communication and feedback and/or debriefing conversations. Existing guidelines for assessing research quality and synthesising findings presume an analysis of empirical research, whereas our task was to synthesise frameworks and recommendations in the form of meta-synthesis [8]. Elements of realist, thematic and meta-narrative synthesis [9] allowed for the analysis of communication frameworks across clinical, clinical supervision and simulation-based education contexts. The process employed an iterative process of framework identification, comparison, synthesis and consensus building, with the expert panel expanding until saturation was reached. Additional literature identified in the review process was incorporated into the model, and the process depicted in Fig. 1 was guided by the SRQR standards for qualitative research [10] .

### Identifying frameworks

Systematic reviews, conceptual papers, and commonly used teaching heuristics were included in our analysis. Given that the nature of the synthesis was designed to build upon existing work, an expansionist rather than reductionist method of accumulating and selecting literature was employed. Therefore, we commenced with the frameworks that had already synthesised the literature from a systematic review, expanded and refined, rather than set a broad search, eliminate and build. The English language literature was screened to identify one author’s (ML’s) starting points using well-known search engines (Pubmed, google scholar CINAHL), search terms feedback, medical education, rubrics guidelines, and models. The purpose of identifying frameworks was to maximise utility rather than focus on comprehensiveness, which was consistent with non-reductionist methods (from the whole “universe” of frameworks), though it was syncretic.

### Creating an initial framework

We selected standard patient communication frameworks using the Kalamazoo consensus statement to synthesise existing literature published by an international panel of authoritative experts [11]. We then tested this against quality supervisor feedback model developed by one of the authors that was based on a Delphi consensus process [12], and this gave rise to an initial consolidated framework for both patient communication and supervisor feedback.

### Selection of articles to test against draft models

Subsequent literature selections were accessed to test them against the framework by utilising existing reviews, as well as searching reference lists and citations of articles and individual databases.

Articles were selected by each expert on a pragmatic basis, and literature was judged useful if it added a new perspective or it was commonly utilised, well known or frequently cited. Identified articles were stored in a common online folder, and a list of key articles that were tested against the current synthesis was maintained. Given that authors were encouraged to test a broad range of formal and informal frameworks, a complete list of non-informative frameworks was not maintained. Useful articles were stored and distributed to the authors online, and they were also subjected to quality assessment, which was not used to exclude articles that were considered methodologically poor because they could still contribute, and it was deemed useful if it added to the draft model.

### Constant comparison

We evolved our draft models by employing a constant comparison process, where each new model was tested against the existing model, and the perspective, assumptions and structure of each model were compared. When the new model added further information, the authors collectively determined if it should be accepted, and if so, a modified model was then adopted.

### Evaluating the quality of included literature

Our review of existing quality standards, including RAMESES [9] and SRQR [10], revealed a substantial overlap among the different EQUATOR standards [13], though this was not the case for the meta-synthesis standards. The EQUATOR standards also did not include certain variables that were deemed important in this context, particularly those related to evaluation and impact. Consequently, the SRQR standards for qualitative research papers [10] were considered, and this was supplemented with considerations associated with the RAMESES standards and educational impact evaluation. An article quality assessment tool was derived by consensus of the research team members based on characteristics of agreed-upon high-quality papers. Nine parameters (perspective explicit, evidence based, reflexivity, iterative development, stakeholder consultation, evaluation performed, scale of evaluation, generalisability and evidence of impact) were then rated from one to three and then summed. The research paradigm is one of communicative action and a post-positivist pragmatic method orientated towards a mutual understanding of the aim of action [14]. The framework used and finding are presented in Additional file 1

### Synthesis of findings

The analytic process utilised multiple methods suggested by [8] that drew on taxonomic analysis (identifying underlying structure and categories) and constant targeted comparisons, which included testing new data against the provisional model; identifying imported concepts (importing concepts from one literature to another) and reciprocal translation (synthesis of related concepts). Common themes were identified and then incorporated into the analysis.

The process was intrinsically based on previous high-quality systematic synthesis efforts by starting with existing frameworks.

### Maximising trustworthiness of findings

The research team comprised experts in the chosen areas of communication (patient communication, supervision and simulation, which included an interest in overlapping areas and were based on existing networks). The authors have a common interest in patient-centred and learner-centred paradigms, the transfer of frameworks to lifelong learning and work in academic health centres. The research team members were specifically chosen to maximise the breadth of experience and ability to identify relevant frameworks employed in their areas of expertise.

A running narrative summary of modifications was kept in a reflective log, and trustworthiness was maximised by employing an iterative process of reflection and a cross validation of the findings. This resulted in a draft framework that was then given to each additional author for sequential modification, cross checking and assessment of credibility.

### Evolution of the analysis

Multiple communication events could have been included in the scope of this study. The initial focus was on comparing patient conversations and providing feedback, though it soon became obvious that the literature regarding simulation was particularly rich, which was within the expertise of the group. It was possible to include other person-centred or performance related conversations, such as debriefing after a critical incident or a coaching conversation, A real possibility of extending beyond medicine to other critical conversations also existed. A pragmatic decision was made to limit the scope to an evaluation of the feedback, simulation and patient-centred conversations. The process continued throughout the manuscript submission process with the incorporation of additional literature and perspective supplied by the manuscript reviewers.

## Results

A total of 14 simulation papers, eight patient interview and nine feedback papers were tested against the framework. Examples of publications that were selected by the expert panel and tested against the framework until data saturation was reached are presented in Additional file 2. Two additional references identified in the review process supported and expanded the existing findings.

### Quality of evidence

Ten clinical communication models were also included. Of these, seven were deemed high-quality models, while the remaining three were found to be moderate-quality models. The major variations included the development of an iterative, reflexive development process that engaged stakeholders. It was possible to evaluate ten of the eleven simulation models, which included four low-quality and six moderate-quality versions. Variations across multiple areas, particularly evaluation, were identified. Seven of nine feedback models were evaluable, and two of those were characterised as having high quality. Evaluations revealed low levels of Kirkpatrick’s pyramid satisfaction and learning [15], though none of the evaluations revealed an impact on clinical behaviours, and no observable relationship was found between utility, popularity and quality score.

### Conceptual structure

**Fig. 2:**
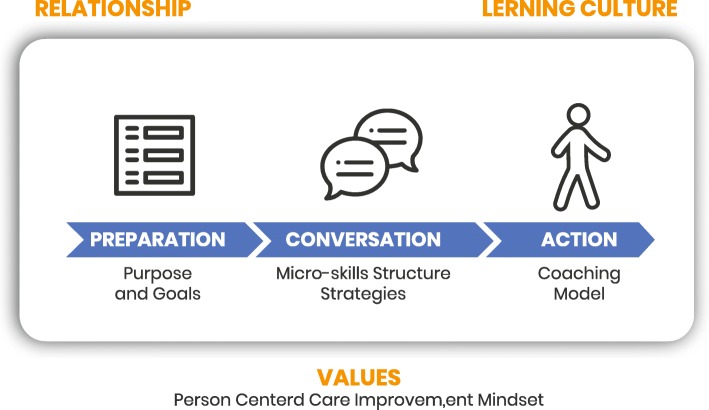
Conversation Structure. Each conversation was constituted by a preparation, conversation and action phase. Preparation was supported by a clear purpose and goal setting. Conversation was supported by micro-skills, clear structure and strategies. Action was supported by a coaching model. The process occurs in an environment determined by values (person-centred care and an improvement mindset), the learning culture and the relationship in question

The available literature had a very unstructured series of recommendations with a mixture of different concepts. We found an underlying structure that consisted of phases, strategies, micro-skills, meta-skills and overarching purpose and goals, though the reviewed frameworks often mixed up these elements. The literature identified from a behavioural perspective tended not to emphasise culture and environment. The conceptual structure is represented in Fig. 2.

### Key themes

The key themes aligned with different ways of viewing these conversations. Conversation as a practical task highlighted the importance of structure, managing the agenda and coaching to achieve change. A mindset of improvement established the goals of improving patient and student outcomes. Conversation as learning prioritises reflection. Conversation as a relationship highlighted the importance of emotion and psychological safety. Viewing conversation from a cultural perspective highlighted the importance of learning culture, while viewing it from a critical perspective highlighted the role of empowerment. The concept of patient-centred care and student- centred learning could be reciprocally translated into person-centred care as a unified concept. A historical trajectory was identified in the literature, which evolved from more paternalistic to more person-centred models, while the different historical trajectory of simulation was found to be a more recent development. These themes played out differently across the conversation phases and with each of the identified strategies.

### Phases

A simple and repeated theme demonstrated the importance of structure. A characteristic method of structuring conversation was divided into a beginning, middle and end. The importance of preparation before the conversation and the follow up actions afterwards were not so consistently recognised within frameworks.

Preparation is recognised as an explicit requirement in the SPIKES framework for breaking bad news in the clinical context, [16] and in the PREPARED framework and end-of-life context, [12] identified the need to organise opportunities to obtain direct observations and allocate the time to provide timely feedback as key characteristics. The London Handbook [17] highlights the identification of the simulation’s learning objectives as a preparatory step. Preparation can be implied in other frameworks, but it is not an explicit feature.

Inconsistent attention has been paid to the follow up and the enaction of change in the patient communication literature (for example, SPIKES does not emphasise this, but a more recent model PREPARED does). A commonly identified theme was the adoption of imported concepts from coaching models. The concept of transferring discussion into action is a crucial one, [18] and it is a particular focus in the coaching models [19]. An explicit item in the definition of behaviours of educators [12] is “educator plans with the learner to review the impact of feedback on subsequent performance”. Older supervisor feedback models have been conceptualised for the purpose of delivering the information rather than enacting change. The need for attention to be paid to learners’ transfer of action into practice is a feature of more contemporary models [20, 21].

Explicitly extending the phases of the conversation to include the time before (i.e., preparation) and the time after the conversation (of following up plans and enacting change) represents an attempt to prioritise two important steps. First, a conversation is integrated into a therapeutic relationship or an educational alliance, and the emphasis then shifts from talking to communication that enables action. A coaching model was commonly used to frame this task.

Although a variety of different structures were proposed, the advantage of a structured approach was apparent [22]. Structure acts as a navigational aid, and it supports the arrangement of conversational elements into sequences that promote the establishment of trusting relationships and reflective dialogue between the parties.

### Strategies

A variety of actions were recommended to meet the goals of the conversations. These strategies were often aligned with the phases of the conversation. For instance, establishing empathy was recommended in the early phases of conversations. The concepts were remarkably similar, although the included strategies and those that were omitted were inconsistent. Those seeking a heuristic were often shorter compared to the more theoretical and inclusive frameworks. We identified a common set of strategies matched to the phase of the conversation, and this is embodied in the acronym EMPOWERS: **E**xpress empathy and emotions, **M**anage the agenda, share **P**erspectives, share **O**bservations, **W**ork together to establish goals, **E**nable, **R**each agreement, **S**ummarise (Table 1).

**Table 1:** Phases of Clinical conversations and associated goals in the Prepares, E.M.P.O.W.E.R.S, Enacts framework

Phase	Goals
Prepares	Sets the scene for a productive conversation
Opening	E – display empathy /address emotions
	M - manage agenda
Middle	P - seek other’s perspective
	O - share own observations
	W - work together on goals (to achieve treatment or close gaps)
	E – Empower the other with commitment to goals and self-efficacy
	R – Reach agreement on plan
Close	S – Summarise issues, goals and agreed plans
Enacts	Follows up and ensures actions occur

Establishing an empathic relationship that allowed for the expression of emotion and management of the agenda was associated with the initial stage of the conversation. Collaboration dominated the middle part of the conversation with a reflective approach to the establishment of each person’s perspective, sharing observations, working together to establish goals, enabling change and reaching a common plan. The final stage of the conversation was dedicated to summarising and ending the conversation and organising follow up to ensure actions occur. Although particular strategies align with particular phases of the conversations, this alignment was not absolute.

#### Express empathy and emotions

Empathy is emphasised in many framework such as R2C2 as a supervision model [20] and PEARLS as a debriefing model [23]. Dealing with the emotional component of clinical conversations is an important task, and all three conversations across three contexts are acknowledged as emotional work. This is most obviously addressed in patient communication models [16, 24], but the emotions for supervisor/facilitator and learner are recognised in feedback models, particularly when learners are invested in the practice being scrutinized [25, 26]. Models of reflection commonly used in simulation debriefing [27] focus on what happened and how learners felt about it, which serve as a reminder that learning is socially-situated.

#### Manage the agenda

Managing the agenda is inconsistently recommended as a strategy. There are two aspects to managing the agenda: negotiating a shared agenda and managing time. A shared agenda is central to the distribution of power and to subsequent stages of shared meaning and shared solutions. Managing the agenda is a goal that is constantly re-negotiated throughout the conversation. Managing time is a goal that usually sits with the clinician or educator, but this can be challenged [28]. Watterson’s framework for simulation-based debriefing emphasises managing the stages of the interview in terms of a beginning, middle and end [22]. Managing time of the interview is a practical skill and prioritising multiple potential topics of discussion in a negotiated fashion is necessary to make the most of the available time.

Criteria for excellence in feedback identified tasks, such as determining the objectives for discussion, comparing efforts to target performance as elements of high quality feedback that fit within the goal of managing the agenda [12] . This same framework also pays tribute to educators’ acknowledgement of the learner’s agenda within the conversation, which may be aligned with the educator’s priority, or it may be disparate, which leads to a negotiation regarding what is important to whom, and how these competing agendas might be managed within a finite period of communication.

#### Share perspectives

The importance of eliciting the patient’s or learner’s perspective is a hallmark of patient- [29, 30] and learner-centred practices in a therapeutic and educational alliance, respectively [31, 32]. In simulation debriefing, eliciting the perspective is expressed as “how did it go” in the London model or “what happened” in Gibbs’ model of reflection [27]. The advocacy-inquiry method particularly emphasises the central role of genuine curiosity about what the other person is thinking, rather than immediate judgement in developing a shared perspective. The learner’s perspective in a feedback conversation becomes self-assessment. Facilitating self-assessment (or broader still, evaluative judgement) is both a process within feedback and a feedback outcome [18]. In the clinical domain, there is an increasing awareness of the importance of a more meaningful elicitation of the patient’s perspective [33]. In the Kalamazoo statement, exploring contextual factors that shape the patient’s perspective, beliefs and concerns and expectation regarding health and illness are emphasised along with acknowledging and responding to this perspective [11].

#### Share observations

The sharing of observations between both sides to ensure that the communication encounter takes the form of a conversation was a common theme [11, 29]. In patient communication, this is reflected in the patient’s history and the sharing back of synthesised information from their history and examination. In feedback (supervision) and debriefing (simulation) conversations, the supervisor/facilitator is encouraged to offer their own perspective, which may challenge or agree with the learner’s judgement of performance or interpretation of events. In the simulation literature, there is an emphasis on judgement and sensemaking serving as keys to the facilitators’ observations. Sharing observations emerges as a recurring theme due to its emphasis on privileging perspectives of communication partners and acknowledging that ‘reality’ is socially constructed rather than definitive and singular. This process aims to create shared observations and thinking, which is consistent with the development of shared mental models [34, 35].

#### Work together to identify goals

Working together may be an implicit value demonstrated through explicit reciprocal offering of perspectives or negotiated outcomes. The educational or therapeutic alliance is expressed as a “partnership” in the PEARLS model in the simulation context. Building a relationship is seen as the core task of patient communication in the Kalamazoo consensus statement [11]. A goal-directed approach is consistent with a coaching model and an improvement mindset, where the goal of each conversation is focused on improving outcomes instead of simply sharing information.

#### Enable

An awareness of the importance of managing the power differential in both clinical and educational conversations is a relatively recent development [36] and a feature of more contemporary communication frameworks [37]. Learner or patient empowerment, or agency, infers a proactive strategy to address this imbalance. Empowerment is a common strategy used in coaching conversations, where the role of the coach or facilitator is to enable the learner to achieve their self-determined goals [38]. Empowerment of patients is a key tenet of the patient-centred care model, and this has received increased focus in clinical communication and medical education research over the past decade [39]. It has been increasingly recognised that many of the thorny issues related to providing appropriate health care, balancing efficacy and toxicities, negotiating futile treatments, promoting healthy behaviours in chronic care and enhancing adherence to treatment plans all require an empowered patient [30, 40].

#### Reach agreement

Negotiating a common understanding of the meaning of what happened (a learner in an educational encounter or a patient’s current situation) and the actions that are required is the core of the communication transaction and follows an agreement on the agenda and observations. Making these understandings explicit and confirming with both sides of the conversation is a crucial step. The strategies presented in Table 1 amount to working through an agenda that is designed to facilitate a common understanding regarding the power in the relationship [41], considering the barriers and enablers of successful action and the strategies developed to deal with these elements. Eliciting a commitment to change is one evidence-based strategy that can be used to enable this [42, 43].

#### Summarise

Summarising is an element in most frameworks examined and emphasises the importance of being able to synthesise and check understanding of all parties in the communication encounter. Its use reflects the conceptual complexity in the field as it can be an essential communication micro-skill that is used throughout the communication, as well as a strategy that is utilised at the end of the conversation. In Kalamazoo, closure is represented as an opportunity to summarise, check understanding and ensure that attention has been paid to the patient’s agenda [11]. Summarising is also an educational strategy that enhances recall of critical conversations through reinforcement and repetition.

### Goals

The overarching goals of a communication framework are either made explicit [12, 21] (as with learner-centred feedback to improve performance) or most often, implied, through certain articulated principles. In both cases, based on the strategies within the model, we have taken an openly ideological stance that the aim of a framework is to direct participants’ towards identified improvements (in the learning context) or ways forward in their care (improvement in health, or maximising quality of life). The centrality of ‘improvement’ and ‘agency’ is reflected in the way that different frameworks have explicitly dealt with issues of learner/patient centredness, power, equity and empowerment.

#### Empowerment

The historical development of the frameworks demonstrates an evolution from more paternalistic models of patient care and supervision to patient-centred and learner-centred models. This is reflected in contemporary patient models such as the Kalamazoo statement, which privileges patient goals and patient participation, whereas with supervision, there is a discourse of learner agency and learner centredness that is related in the concept of the educational alliance. However, the presented models are presented from the viewpoint of the supervisor and very much focused on the responsibilities of the supervisor. An exception to this is the “PROMPTED” model of, [41] where the model is explicitly written from the point of view of the learner and focuses on their actions. The simulation literature has emerged from a contemporary perspective and has adopted the concept of facilitation by using a model where the group is empowered and the educator’s role is facilitatory rather than instructive or didactic [44].

Supervision has come from a paternalistic model of apprenticeship, but more recent literature adopts the perspective of self-determination theory with the need for competence, autonomy and relatedness. In particular, the potential for informed self-assessment or evaluative judgement [45] has emerged as a key capacity for learners to develop (external feedback provided by others of course helps to hone this capacity for making judgements about quality of work). These principles of placing the ‘learner’ at the centre also underpin coaching practices [19] and motivational interviewing [46]. Coaching has been a stronger influence in the simulation literature than it was in older feedback models, although more recent models such as R2C2 have been explicitly built around coaching principles. Coaching has also made inroads into continuing professional development [47], doctor-patient communication (particularly in relation to drug and alcohol abuse), chronic disease and long term behavioural change [42, 48, 49]. It is recognised that translation of concepts or knowledge into behaviour change requires engagement, and opportunities to tackle a subsequent related task.

#### Psychological safety

The issue of an imbalance in power along with the emotional nature of difficult conversations highlights the issue of psychological safety and the potential for harm. Creating a safe environment is a feature of the simulation literature, and it is assumed within the clinical conversation (i.e., implied as part of the therapeutic alliance with the acknowledgement of the role of trust in communication), but the issue of generating open and productive spaces for learning conversations in the supervision literature is not well understood [50]. Historically, the need to maintain social harmony has been reflected in the focus on models like ‘the feedback sandwich’, which requires a balancing of negative and positive information [51]. Well described characteristics of educator behaviours improve the effectiveness of feedback, such as holding the learner’s best interests at heart and using reciprocal vulnerability as a way to promote open disclosure and learning [52]. However, these characteristics or qualities may be difficult to enact due to the strongly embedded rituals of ‘educator telling’ in feedback practices. The RC2C explicitly acknowledges feedback as a relational activity and emotions are acknowledged rather than bypassed by ‘disguising’, softening or ‘sugar coating’ rituals [20]. Creating psychological safety requires an authentic awareness of the vulnerability of the individual and a flexible approach to tailoring feedback within the boundaries of what can be safely heard by the listener, whether they are a cancer patient not ready to hear that they are dying, or a learner who is not able to hear the “full story”. The goal is more than truth telling. It includes appropriate action, and this requires meeting the other where they are and safely helping them move in the direction required.

#### Encouraging reflective practice

The importance of reflective practice is built into the supervision and simulation literature through structures of self-reflection in both conversations. The role of reflective practice within a patient conversation is not so well articulated. Giving that a patient is responsible for their own management, and encouraging them to reflect on how they are going is a significant shift. Asking patients and clinicians to reflect on their perception regarding how a therapeutic relationship/alliance is going is also a paradigm shift, which requires empowering the patient to raise concern or address unmet needs.

### Communication micro-skills

A variety of recommendations have endorsed good communication principles, and (in line with the concept of counselling micro-skills) we have grouped these as communication micro-skills. Active listening, effective use of questioning and non-verbal interaction appear, almost universally, in literature on various conversation techniques. The patient-centred interview technique classifies communication skills into those that are non-focusing and focusing [29]. Non-focusing techniques include silence, non-verbal encouragement and neutral utterances, whereas focusing techniques include reflection, echoing, open-ended requests sign posting (explicitly naming structure) and summarising. Active listening is emphasised [53] including strategies to respond to emotional cues, such as Name, Understand, Respect, Support and Explore (NURSE) [54]. Some communication skills are emphasised within specific literature (such as advocacy-inquiry), but they have the potential to inform other conversations. Communication micro-skills are summarised in Table 2.

**Table 2:** Groupings of communication skills in the literature as applied to patient communication, feedback conversations and simulation debriefs

Grouping	Strategies	Context (References)
Counselling Micro-skills		
(1) Attending behaviours	Eye contact, vocal qualities, verbal tracking.	Patient [55]
(2) Body language	Squarely face, Open posture, Lean, Eye contact, Relaxed(SOLER)	Patient [56]
	Sit at an angle”; “Uncross legs and arms”; “Relax”; “Eye contact”; “Touch”; “Your intuition” (SURETY)	
(3) Questioning	Open versus closed questions	Simulation [57]
	Encouraging, Summarising, paraphrasing	
(4) Promoted self-reflection		Patient [58] Feedback [37] Simulation [27]
(5) Active listening	Name, Understand, Respect, Support, Explore	Patient (Back et al. [54])
(6) Signposting	Name structures to facilitate navigation through conversation.	Patient [59]
Patient centred interview technique		
(1) Focusing	Silence,	Patient Fassaert et al. 2007 -[60]
	Non-verbal encouragement and neutral utterances	Simulation [44]
(2) Non focusing	Reflection, echoing, open ended requests and summarising	
Meta-skills		
Cognitive appraisal -Cues	Picking up and responding to patient cues	[21]
Cognitive appraisal -Barriers	Uncovering and resolving barriers to communication	
Mindfulness	Full immersion in the moment	[24]
Community orientation	Recognising and acting upon the importance of community supports such as family (for patients) or peers (for learners)	
Team orientation	Recognising the importance of the team and acting accordingly	
Simulation techniques		
(1) Advocacy-inquiry	Advocating a particular interpretation -combined with genuine curiosity as to whether that interpretation is correct.	Debrief [61]
(2). Group techniques (Debriefing)	Team guided self correction	[44]
	Circular questioning	

### Communication meta-skills

Two frameworks identified communication skills that could be considered meta-skills, or skills that enable other skills. The COMSKIL framework identified cognitive appraisal as a key concept that extends beyond communication micro-skills [21]. This emphasises the importance of cognitive appraisal of patient cues and barriers as enabling communication. The COMFORT framework identified mindfulness, a team perspective and family orientation as enabling skills [24]. From a learner’s perspective, an equivalent recognition would include the importance of peers and the healthcare team in enabling learning.

### Similarities and differences between conversations and context

This analysis demonstrates the overwhelming similarities in the structure, goals, strategies and themes used for different conversations: patient communication; supervisor feedback and simulation. However, there are differences in the way these elements play out and the responsibilities of each party at each phase of the conversation.

There are practical differences between the conversations. In patient communication, a physical exam may be inserted into the conversation. In workplace learning, there are similar decisions about how to incorporate the observed practice and feedback, whereas the processes are distinctly separate in simulation, which is usually conducted as a team activity.

The role of clinical reasoning is different within the learner-centred conversation in that there is a goal of teaching clinical reasoning, and learners will start with “patient-like” reasoning and gradually incorporate clinical reasoning. This raises the question of how much clinical care should include teaching clinical reasoning.

The role of values is also different. It is accepted that patient values might be quite varied, for example, the impact of disability on the value attributed to life extension. However, learners share a common set of values, and incorporating these values is part of navigating through a community of practice.

#### Preparation

In the preparation phase in the clinical setting, both parties must review the information available to them. In the supervision setting however, the preparation is contested. For example, in a learner-centred implementation, the learner selects the activity to be observed and organises a time for review. Conversely, in a supervisor-centred implementation, the supervisor drives the nature and timing of activities. In the simulation setting, the activity is typically highly structured, and the simulation team will own the preparation.

#### Opening

The strategies related to opening focus on feeling empathy, establishing rapport and managing the agenda. There is variability in opinions regarding whether the initial process is called empathy or rapport. Rapport is a broader term that includes multiple strategies for enabling communication and includes empathy as a central focus, while strategies that put the parties at ease, such as the use of humour, can also build rapport. The responsibility for empathy and rapport sits with the clinician and supervisor in their encounters, although the responsibility of the patient and learner to have empathy for others is an important consideration. In simulation, the facilitator has a role to ensure that the group owns empathy with each other and creates rapport. In the clinical encounter and supervision, the agenda is contested according to patient-centred or learner-centred implementation, whereas the overall agenda is relatively pre-determined in simulation. The culture in simulation considers the agenda as an element to be determined by the group as the simulation develops.

#### Middle

The strategies in the middle phase are related to POWER (perspective, observation, working together, empowering and reach), establishing a mutual understanding of what happened and working together to reach a goal. In the clinical and supervision settings, this process is contested with the balance of how this plays out determined by other-centredness. In simulation, the facilitator’s role in the situation is designed to leave ownership and power with the group. In each case the underlying process is the same.

#### Close

The end phase is about summarising a shared perception and plan along with checking on the agreement. This is traditionally the responsibility of the clinician, supervisor or facilitator, although it could be deferred or delegated to the patient, learner or group. It is particularly important to clarify the immediate next steps and final documentation.

#### Enact

It is typically the responsibility of the clinician, supervisor or facilitator to determine what follow up is required and the responsibility of each party as it relates to enaction.

### Alternative perspectives

A segment of the literature has identified addressed feedback conversations from different perspectives other than structure and strategies. The RIME rubric is a framework for progression that can be used to structure feedback by setting the standard as reporter, integrator, manager or educator. It provides different functions within a workplace assessment that are structured hierarchically [62]. The idea is that different feedback is given when a person functions in the role of manager rather than educator. Ramani et al. offered a different perspective that focuses on the content of the feedback classified according to the Johari window, which is a classification of self-awareness into the known, the unknown, the hidden and the blind [63]. These approaches share an interest in an underexplored issue in these conversations, namely how the agenda is selected and from what perspective is the problem approached. The identification of alternative perspectives highlights this choice.

## Discussion

Clinicians are expected to be effective communicators and possess a range of core transferable communication skills across a range of contexts. This is enshrined as a key competency in the CANMEDS framework, which has been widely adopted in medical education [64]. Patient communication and supervisory feedback/debriefing represent two broad applications of communication. Simulation feedback is an example of a feedback scenario where the activity is well structured, and the unit of observation is often a team rather than an individual. It has different historical antecedents and remarkably separate literature.

The choice of an appropriate analytical method to compare and synthesis structure and “communication frameworks” raised many significant issues, and existing guidelines, such as those on the EQUATOR network, did not fit the goals of this research.

The meta-analysis model of quantitative observations presumes the identification of a “complete universe” of literature regarding a defined topic, a filtering process based on quality and an unbiased synthesis of findings. A similar process has been utilised in existing approaches to the synthesis of qualitative observations. The process can be conceptualised as the “distillation of a concept”, and it has been derived as fit for the purpose of synthesising observations to guide theory.

We found this process was not fit for the purpose of synthesis concepts or frameworks to guide practice. Conceptualisation of utility in education suggests that a useful framework would depend not only upon the development process, but more importantly, on acceptability, feasibility, educational impact and impact on practice. Given that there is little data on any of these endpoints for most communication frameworks, we deemed the “Gold Standard” for assessing these frameworks is expert consensus.

In this setting, a rigorous process would build upon existing expert consensus and bias any synthesis towards existing frameworks with a broad consensus, broad acceptance or those that did have evidence to support them. We therefore utilised a process that started with the best available models (by expert consensus) and iteratively tested the synthesis model against the best available alternatives. Unlike methods based on the model of a qualitative synthesis, the method could be imagined as aggregative “crystallisation” from a nidus concept rather than distillation. Rigour in these circumstances is derived from the concept of data testing until saturation is reached, rather than analysis of a complete data set. Bias is welcomed and incorporated into the process rather than eliminated.

We therefore conducted a meta-synthesis of the literature that encompasses communication within clinical conversations, supervision and simulation, which seeks to elicit common structures and recommendations. We chose these dialogues as critical, performance-centred and action-directed “conversations” that are core to the mission of patient care and clinical educators. Our analysis revealed a great deal of similarity between these literatures, even though the specific recommendations and acronyms can differ significantly in practice.

This analysis has elicited an underlying structure that is common to these related conversations, which has clear phases, including preparation, conversation and action. Each conversation has a beginning middle and end, each phase is supported by key elements and preparation is supported by a clear purpose and goals. Translating this into action is supported by coaching for change management. The conversation itself is supported by a clear structure, strategies that align with goals, and communication micro-skills. A set of meta-skills enables other skills, including cognitive appraisals and mindfulness. The whole process is informed by the same values that inform the purpose and conduct of the conversation, and it takes place in the context of a relationship and a clinical environment.

The connection of the conversation with the underlying relationship through preparation before the conversation and following up afterwards is relatively poorly developed. The goals at the beginning of a conversation revolve around creating an empathic relationship, building rapport and managing the agenda. The goal at the end of the conversation is to consolidate agreement that will lead to action. The middle of the conversation is where a mutual understanding and plan are co-constructed. The strategies employed here are designed to negotiate a common understanding of what has happened, what it means, and what must be done through a process of shared decision making. Consequently, checking understanding and structuring explanations to aid recall are particularly important skills at this stage.

The identified strategies can be summarised by using the mnemonic EMPOWERS: **E**xpress empathy and emotions, **M**anage the agenda, share **P**erspectives, share **O**bservations, **W**ork together to establish goals, **E**nable, **R**each agreement, **S**ummarise.

### Comparison with other literature

Other literature highlights were compared with the differences associated with our analysis, which included methods, findings and implications for practice. This work contributes to the broader discussion regarding qualitative data synthesis. Frameworks and heuristics are different types of data to analyse and synthesise compared to other qualitative data sources. They are a form of synthesis, which makes this analysis fit within the broad grouping of meta-synthesis. The aim was to expand and critique existing frameworks by providing an iterative process and starting with the best available data synthesis. We therefore developed a novel expansionist (i.e., narrow to broad) recruitment of literature rather than a reductionist (i.e., broad to narrow) search strategy to achieve our research aim. This qualitative synthesis approach adds to the repertoire of available methods and emphasises the need to develop standards that are fit for the purpose.

The taxonomy we developed through the synthesis process has many similarities to the COMSKIL model [21]. For instance, it shares a common concern with clearly distinguishable skills, processes and strategies and the development of tasks out of common skills. It also shares a concern with action that aligns with goal, plan and action theories. These findings consolidate published guidelines regarding how to conduct these conversations. A systematic framework helps to identify those elements that have been chosen and those that have been omitted. A comparison of these conversations highlighted the similarities and differences in the literature related to these closely related conversations and the potential for “imported concepts” from one literature and community of practice to inform the others. It also particularly highlighted the utility of person-centred communication as a term to connect concepts of patient-centred care and student-centred learning.

The “critical” approach to analysis particularly highlighted the issue of power and empowerment in conversations. Definitions of these key concepts relating to power are summarised in Table 3. These strategies purposively redistribute power within the relationships from historically imbalanced “paternalistic” relationships towards more equal partnerships: from the concept of truth and knowledge being owned by the powerful; to a reality where the experiences of the patient and learner are valued in their own right, and truth is co-constructed. The focus shifts from “telling” to the consequences, which serves as an acknowledgement that patients and learners have always had the power to be “non-compliant” with strategies they did not help create. These historical and cultural changes are reflected in the change in frameworks over time. They are reflected in models of communication that consider the diverse functions required of a communication encounter as relational, emotional events that connect information, decisions and action, which have the potential to empower patients [76]. Power, empowerment and person-centred care were central themes in the literature examined as part of this study. Other related issues include the importance of a coaching mode to enable other-centred care that creates a safe psychological environment and a link to change.

**Table 3:** Key Concepts and Definitions

Appropriateness. The consideration that a method of communication or research is fit for the purpose for which it is intended. It implies selection from alternative methods driven by purpose.	
Behaviourism. A worldview that assumes a learner responds to environmental stimuli in a predicable way. The learner starts off as a clean slate (i.e. tabula rasa) and behaviour is shaped through positive reinforcement or negative reinforcement^]^ [65]	
Critical Theory. A view that theory is historical, subjective, and a part of society. Critical theory is in this regard a highly reflexive enterprise” it is also concerned about the consequences of asking these questions [66]..	
Culture. Consists of the values, beliefs, systems of language, communication, and practices that people share in common and that can be used to define them as a collective. This includes cultures brought by individuals from their experience, as well as professional, organisational and national cultures [67].	
Discourse. A collection of conversations, which is “a coherent system of meanings, realized in texts, which reflects on its own way of speaking, refers to other discourses, is about objects, contains subjects and is historically located [68]. The implication is that different discourses look at a problem from different perspectives and with different answers.	
Empowerment. A social action process that promotes participation of people, organizations, and communities in gaining control over their lives in their community and larger society [69].	
Learner-centred teaching. A method that places the learner at the centre of goal setting, selection of learning activities, is based upon a coaching model and depends upon the relationship with the teacher; the educational alliance [32].	
Paradigm. A universally recognized scientific achievements that, for a time, provide model problems and solutions for a community of practitioners [70] It is a way of thinking which is one of the limits on how a problem can be understood.	
Patient- centred care. A model of care which places the patient at the centre of goal setting; seeks to understand problems from the patients perspective, is holistic, is based on a coaching model and depends upon the quality of the relationship between the clinician and patient- the “therapeutic alliance” [71, 72].	
Power. The ability of an individual, group, or institution to influence or exercise control over other people and achieve their goals despite possible opposition or resistance. The contribution of Michael Foucault [73] however has been to draw out the relationship between knowledge and power and to see power relations as constitutive of all fields [74]. This leads to a critical approach questioning the role of power relations in every discipline [75].	
Taxonomy. A classification system based on underlying observable structures or themes. Developing a taxonomy is a form of qualitative inquiry.	

The authors are not suggesting that EMPOWERS should become another mnemonic to replace SPIKES or PEARLS. Communicators should use frameworks that suit their context, goals and environment. The utilisation of any framework has limitations and has been criticised as being reductionist and behaviourist [77, 78]. Any attempt to force an “organic” entity, such as a difficult conversation, into a box risks inhibiting the creative process, which is required for truly skilled, individualised and person-centred communication. For example, it has not been suggested that empathy is only important in the opening of a conversation, merely that it is an important to establish empathy early.

This framework has not been proposed as a rigid structure. Expert clinicians learn, remember and are adaptable [79] when it comes to how they incorporate these structures into practice [80]. Master communicators reassemble frameworks on the fly and respond to individual circumstance in creative ways that are opportunistic, personalised and authentic.

Significant benefits are realised from the development of a comprehensive framework. Such a framework aims to expand the toolbox of strategies and the skills available to clinicians beyond their favourite acronym. A critical and comparative approach also identifies the assumptions behind specific frameworks and the strengths and weakness of a preferred framework. Regardless of the framework used, making structure memorable and explicit plays a critical pedagogical role.

Practice related to critical reflection on the strengths and weaknesses of any chosen framework is designed to guide communication encounters. The framework provides a starting point and a common language for a conversation about how we can better integrate teaching of different communication skills in a spiral curriculum. Clear examples of this include taking the opportunity to promote common micro-skills across scenarios and highlighting the importance of planning and follow up.

The framework aligns ideologically with the priorities of our time to enable and encourage person-centred care and education. Another strength, of a common framework, is that it promotes a congruence in our behaviours as clinician and educator. Person-centred care is an overarching concept that applies a consistent “way of being” in our relationships with patients, students and peers. It also aligns closely with the concept of compassion in our relationships with others and promotes the value of consistent role modelling of this core practice.

This analysis addresses our aims of presenting a systematically derived framework and recommendations that apply to clinical conversations across different contexts, and the importance and the repeated teaching of those common micro-skills and goal-directed strategies is emphasised. There is also a focus on the commonality of structure, partnership, empowerment and action as unifying communication strategies, and the trustworthiness of the analysis is maximised due to its iterative reflective nature and the breadth of the authors’ expertise.

The analysis has obvious limitations that may be categorised as methodological and epistemological. The methodological issues relate to the trustworthiness of the findings. The expansionist approach to the finding of frameworks, which begins at the centre in existing frameworks, has the advantage of incorporating existing synthesis methodology, though it biases the analysis of existing concepts. The inability to meaningfully measure the utility of frameworks makes the assessment of quality problematic. There is a risk that other existing models may improve or challenge the framework, though it is minimised by performing testing until saturation is reached. However, the possibility that such models exist cannot be dismissed. The trustworthiness of the findings does not relate well to concepts imported from quantitative data analysis around the completeness of the identified literature, but rather with concepts from qualitative inquiry regarding coherence, rigour, reflexivity and consensus. These relate to the expertise and insightfulness of the panel and the iterative process of finding a consensus within a community of practice. We have explicitly identified the value we place on person-centred care and an improvement mindset in the analysis, and it is important to note that a different panel representing a broader range of cultures or contexts may come up with different priorities.

The epistemological limitations relate to individual variations in the understanding of concepts such as empathy or power, the predominance of the behaviourist paradigm within the literature examined and the limitations of using any framework which is always a form of representation of a phenomenon. An existentialist approach may consider the impact of who the people in a person-centred approach “are”. This is both a contextual issue of each participant’s epistemic stance, attitudes, past experiences and preferences and an existential issue as to who people think they are and how their roles are perceived to intersect or otherwise interact (e.g., practitioner or educator). Whether a practitioner is person-centred and whether they bring their “best self” to the conversation may be more important than the heuristic they follow. Similarly, a behaviourist approach does not acknowledge “who” the recipient is. Consequently, there is a need for clinicians and educators to personalise these skills and strategies so they can communicate authentically with the recipient with the aim of working towards the recipient’s goals.

The assumption that both parties in a communication are acting from a common epistemological framework is another example. For example, if a student/patient has a different understanding of what power or empathy means to their supervisor/clinician, conflict is more likely to occur, and a common understanding of key concepts is thus central to avoiding it.

The behaviourist approach also tends to underestimate context, particularly the influence of culture and relationships, on learning. There have been exceptions where learning culture was explicitly addressed [81], and there is an extensive literature on the importance of learning culture [82–84].

Similarly, including the importance of one’s family or team in the COMFORT framework [24] highlights the existing frameworks’ individualistic perspective.

Similarly, employing a reductionist approach, any communication will focus on the similarities between conversations rather than their differences. A philosophy of difference [83] might emphasise the difference in conversation with a listener based on who the individual is, the level of insight they show, their state of emotional arousal, and/or whether an other-centred approach is preferred. Frameworks constructed in this manner would look very different. An example of differentiating an approach to different “phenotypes” from the simulation literature is presented by [84].

Each of the steps summarised by a letter in EMPOWERS, PREPARE or PEARLS is a complex and rich step must be unpacked. “Perspective” is not just about the listener understanding the perspective of the other person. On the contrary, it is about the process of self-reflection and insight (or lack thereof) that reveals itself in the perspective taken. Empathy is not just about feeling connected and doing something about it. It is a part of a whole process of building rapport and relationship. Inevitably, the process of reducing complex conversation to frameworks and mnemonics requires them to be enriched again in application through the depth of understanding brought to the task and the context that makes up the task.

## Conclusions

Conversation is at the heart of health. How we think about dialogue and teach these skills are critical to healthcare, which remains human amongst rapid developments in technical care. Communication is the common pathway in all that we do. This analysis of published communication models across three distinctive but related contexts brings together a long history of research and speculation about communication. The values that underpin models across contexts are based on person-centred care and an improvement mindset. We may find that highlighting opportunities for translating communication approaches across clinical and educational contexts guides our practice towards ‘improvement’ and brings together more minds to expand the communication research agenda.

## Supplementary information

**Additional file 1.** Evaluating the quality of communication frameworks.

**Additional file 2.** Summaries of individual publications included in the review.

## Data Availability

All data generated or analyses during this study are included with the published article.

## Abbreviations

EQUATOR: Enhancing the quality and transparency of health research
NURSE: Name understand respect support explore
PEARLS: Promoting excellence and reflective learning in simulation
PREPARED: Prepare relate elicit provide acknowledge realistic encourage document
R2C2: Relationship reactions content coach
RAMESES: Realist and meta-narrative evidence syntheses: evolving standards
RIME: Reporter integrator manager educator
SPIKES: Setup Perception invitation knowledge emotion summarise
SRQR: Standards for reporting qualitative research
