# Supplementary material for: Finding common ground: meta-synthesis of communication frameworks found in patient communication, supervision and simulation literature
Source: BMC Med Educ. 2020 Feb 11;20:45. doi: 10.1186/s12909-019-1922-2 (PMC7014645; doi:10.1186/s12909-019-1922-2)
Supplement: Supplementary file 1 — Additional file 1. Evaluating the quality of communication frameworks. [file 12909_2019_1922_MOESM1_ESM.zip › Finding common ground R appendix2 FinalR3.pdf]

APPENDIX 2:

Table 1: Summaries of individual publications included in the review (a) Simulation models (b) Patient interview models (c) Feedback models and (d) identified in review

Model name/ Author	Context/ Target audience/ Publication type/ Description	key elements of framework	Structure	Conversational goals addressed #	Strategies proposed ##
**(Cheng et al. 2016)**	Focus on learner centred approach	Focuses on Reactions and Analysis phase	Identify learner agenda<br>Prioritise content<br>Promote learner self assessment<br>Explore rationale<br>Close performance gaps<br>Manage transtions/Manage time<br>Identify key messages	MPOWERS	AL, NVC, OE,
**ALOBA –Silverman 1996 Reference (Chowdhury and Kalu 2004)**	Supervision / Multidisciplinary/ Critical review of existing models, described by authors as an agenda-led outcome-based analysis.	Rehearsal of skills to achieve outcomes;<br>Facilitator closes and summarises	Learner set goals;<br>Facilitator praises achievements and introduces theories;<br>Rehearses skills	POWES	Not addressed
**Universal Student-Centred Interview**	Supervision and Simulation Multidisciplinary/	"Manage agenda proposed as a 'preview' of the conversation	Preparation,<br>Open, Middle,	E,M,P,O,W,E,R,S	AL, NV, OE, AI

**Framework (Waterson 2013)**	Training manual/ Framework supports supervision conversations in a range of contexts including orientation, workplace assessment and feedback; following up critical incidents and performance management; delivering bad news to students. Delivered as a simulation course.	assuming supervisor/debriefer addresses pre-determined learning or other objectives. Preview outlines a structure for the conversation, includes a summary of key events/issues and sign posting what will be addressed	Preview end, End		
**GAS (Phrampus and O'Donnell 2013)**	Simulation / Multidisciplinary / Structured debriefing model; Gather, Analyze, Summarize in an operational acronym (GAS).<br>Highly structured with specific goals, actions and timeframes for each phase.<br>May use phased domain debriefing i.e. group teamwork then separated clinical debrief for each profession	Gather: learner reactions<br>Recap by team for shared mental model (25%)<br>Analyze: learner-centred reflection and analysis of actions. Facilitator observations and questions to identify performance gaps. (50%)<br>Summarize: learning points revisited / reviewed. (25%)	Gather<br>Analyze<br>Summarize	E,M,P,O,W,S	AL, NV, OE
**3D (Zigmont et al. 2011)**	Simulation / Multidisciplinary / Structured Debriefing model Diffusing, Discovering and Deepening.<br>Authors note relevance to	Feelings included in Diffuse (2nd stage)<br>Ground rules; Format expectations;<br>Explore/Address emotions;	Prebrief ground rules<br>Diffuse<br>Discovering<br>Deepening<br>Summarise	E,M,P,O,W,E,R,S	AL, NV, OE, AI

	supervision in addition to simulation	Needs analysis; Discovering phases uses reflective principles to elicit and share and shape mental frames;<br>Prompt individual to apply new knowledge to practice;<br>Summarise key learning points;<br>Recap			
**Diamond (Jaye et al. 2015)**	Simulation / Multidisciplinary / Structured debriefing model with script: three phases presented in a diamond shape to indicate relative timeframes with clear transitions between each phase.	Feelings de-emphasised as culturally not required in UK; Clarify what good performance is; Facilitate self-assessment; Provide opportunities to close the gap; Include discussion of feedback with peers and supervisors; Elicit feedback on process to aid teaching; Use feedback to improve teaching	Description<br>Transition: clarification Analysis<br>Transition: reinforce agreements<br>Application	M,P,O,W,E,R,S	AL, NV, OE
**PEARLS (Eppich and Cheng 2015)**	Simulation / Multidisciplinary /<br>Structured debriefing model with script. Four phases: reactions, description, analysis, summary.	Integrated model considering context-specific factors influencing the choice of approach to analysis phase (more learner or more facilitator directed), including time available, whether learners' rationale for action is clear, and whether the learning objective/performance gap are	Reactions<br>Description<br>Analysis<br>Summary / application	E,M,P,O,W,E,R,S	AL, NV, OE, AI

		related to knowledge, skills, or behaviours.			
**AAR (Sawyer and Deering 2013)**	Simulation/ Multidisciplinary / Structured debriefing model / Seven phase After Action Review (AAR) of performance based upon the US army debrief model. Uses acronym DEBRIEF	Explicit review of learning objectives and expected performance against a standard. Shared mental model of critical aspects of case Identifying what and why reveals underlying mental model and facilitates change. Prompt to what would do differently next time	Define the rules Explain learning objectives Benchmark performance Review expected actions Identify what happened Examine why Formalise learning	M,P,O,W,E,R	AL, NV, OE, AI
**SHARP (Ahmed et al. 2013)**	Supervision / simulation / Surgical trainees (potential wider application) Pocket tool to focus feedback on learning objectives established prior to case. Aims to make feedback specific and learner centred. Designed as a brief reminder	Learner centred – objectives set by learner. Agenda is managed by following tool that both learner and supervisor have. Observations based upon what the learner set out to do and feedback specific to that. Plan for next time articulated. Feelings not included. Summary of learning not specified	**S**et learning objectives (before case) with learner **H**ow did it go (after case) – compared to expected **A**ddress concerns – what didn't go well **R**eview learning points – met objectives? **P**lan ahead	M,P,O,W,R	AL, OE
**OSAD (Arora et al. 2012; Imperial College London**	Simulation / Multidisciplinary / debriefers Objective Structured	Rates debrief in areas of: Approach / Learning Environment / Engagement /	Open: environment & approach Middle: reflection,	Components of "good" rating scale includes	AL, NV, OE

**2010)**	Assessment of Debriefing (OSAD) tool.<br>Used as a how to guide for novice debriefers, rating tool of feedback quality and research tool to identify if intervention to improve debriefing is effective	Reflection /Reaction / Analysis / Diagnosis / Application<br>Provides anchored rating scale with descriptors of poor, intermediate and good applications. Emphasis on learner centeredness, targeting behaviours that can be changed and application to practice. No diffusing of feelings prior to reflection.	reaction, analysis and diagnosis<br>End: summary and application	E,M,P,O,W,E,R,S	
**Gibbs (Husebø et al. 2015)**	Simulation / Multidisciplinary / Review of Gibbs' Reflective Cycle theory as applied to debriefing in simulation	Six stage reflective cycle with each stage informing the next. Provides script for each stage. Original model for self-evaluation and reflection so does not emphasise facilitator observations<br>Description / Feelings / Evaluation / Analysis / Conclusion / Action Plan	Open: case description and feelings about case<br>Middle: evaluation and analysis of performance, drawing conclusions<br>End: Action plan	E,M,P,W,E,R	AL
**Debrief with Good Judgement (Rudolph et al. 2006)**	Simulation / Multidisciplinary / Structured debriefing model / three phase model with an emphasis on debriefing stance.	Reactions phase for learners to "blow off steam" and provide insight to debriefer.<br>Demonstrate respect for learners through using topics raised in reactions phase as part of debrief. Signpost topic changes.	Reactions<br>Analysis<br>Summary	E,M,P,O,W,E,R,S	AL, NV, OE, AI

	Analysis phase four step process of observe, feedback, investigate and close performance gap. Synthesises and augments improvement suggestions with evidence and experience. Codify insights into “rules of thumb” to improve practice.	

Table 1 (a). Simulation models

#The conversation goals addressed from the prepare-EMPOWER-enact model are represented by the appropriate letter. See manuscript for details Table 1.

The strategies proposed are listed with the abbreviations: Active listening (AL); Non-verbal strategies (NV); Observation-based Enquiry (OE); Advocacy Inquiry (AI). See manuscript for further details.

Model name/ Author	Context/ Target audience/ Publication type/ Description	key elements of framework	Structure	Conversational goals addressed #	Strategies proposed ##
**-PREPARED (Clayton et al. 2013)**	End of life discussions analysis of 22 workshop participants	Detailed observable descriptors of specific behaviours to facilitate assessment. Specific content requirements , less emphasis on structure, more on method	Prepares<br>Relates<br>Explores<br>Provides<br>Acknowledges<br>Emotion and concerns<br>Encourages questions<br>Documents	EPO	Encourages questions<br>Anticipates and Addresses fears and concerns<br>Check understanding
**Scars(Brighton et al. 2018)**	End of life discussions analysis of 886 workshop participants	Focus on clarity and compassion framework elaborated with a workshop discussion	Setting, communicate with kindness, ask respond and reflect, summary and plan	Prepare<br>EMPORS	Not elaborated within manuscript
**(Keller and Carroll 1994) E4**	Four tasks of clinical communication	Structure not emphasised focus on patient education	Engage<br>Empathise<br>Educate<br>Enlist	E M O W E	Open ended questions , non verbal , acknowledgement, self disclosure
**(Kurtz and Silverman 1996) Calgary Cambridge**	Commonly used framework for teaching patient interviewing	Content v process<br>Providing structure and<br>Building the relationship<br>Patients perspective and biomedical perspective<br>Achieving a shared	Initiating session<br>Gathering information Physical examination<br>Explanation and planning Closing the	Preparation<br>M P O W E R<br>Follow up	70 core Process skills open to closed cone, active listening, faciliative reposne , cues, clarification

		understanding	session		, internal summary time-framing
**Kalamazoo (Makoul 2001)**	Consensus amalgamation of 5 commonly used patient communication models	7 tasks. Each task associated with desired behaviours	Build a relationship<br>Open discussion<br>Gather information<br>Understand the patient's perspective<br>Share information<br>Reach agreement on problems and plans<br>Provide closure	E POWRS –<br>Follow up	AL, open-closed, encourage participation as desired, personal connection , identify resources
**(Novack et al. 1992)(BIC)**	Specific behaviours constitute an interview checklist of 32 skills or behaviours	Separate stages and types of skills. Types of skills: facilitation; relationship; patient education.	Stages: opening; Information gathering; closing.	EMPO W<br>Follow up	Support, respect, acknowledges emotions , NVC , summarise, open-closed

**(Smith et al. 2000) Patient- centered Interview**	**Method evaluated in RCT**	**Divides into 5 stages. Describes tasks associated with each stage**	**Setting the stage<br>Chief complaint<br>Non focused interviewing<br>Focused interviewing<br>Transition to Doctor centered**		
**SPIKES (Baile et al. 2000)**	Breaking bad news		Setup<br>Perspective<br>Invitation<br>Knowledge<br>Empathy<br>Summary	Prepare<br>EMPORS	Assess information preferences, empathetic statements , exploratory statements, validation

Table 1(b) Patient Interview models

#The conversation goals addressed from the prepare-EMPOWER-enact model are represented by the appropriate letter. See manuscript for details Table 1.

The strategies proposed are listed with the abbreviations: Active listening (AL); Non-verbal strategies (NV); Observation-based Enquiry (OE); Advocacy Inquiry (AI). See manuscript for further details.

Model name/ Author	Context/ Target audience/ Publication type/ Description	key elements of framework	Structure	Conversational goals addressed #	Strategies proposed ##
**(Milan et al. 2006)**	Feedback based on clinical communication (using "PEARLS" –not simulation model)	Change model with loose structure and coaching strategies	Loosely structured s	E	Partnership<br>Empathic understanding<br>Apology for barriers<br>Respect values and choices<br>Legitimisation<br>Support correction
**(Hesketh and Laidlaw 2002)**	Deal wiih formal and informal feedback	Mixture of stages and strategies	Prepare<br>Respect<br>Self-assess<br>Observations<br>Solutions<br>Check agreement	Prepare<br>PO	Non-judgemental language, respect, engagement, specific examples
**(Rudland et al. 2013)**	Student centered model not a conversation structure	Interaction between structural elements<br><br>Student characteristics of being responsive, receptive and reflective	Student characteristics<br>Contextual factors<br>Supervision characteristics and quality of feedback	W	Precise<br>Relevant<br>Outcome based<br>Measurable<br>Possible<br>Time determined<br>Encouraging<br>Descriptive

**Pendletons rules 1984 referenced in (Chowdhury and Kalu 2004)**	Feedback supervisor facilitator or supervisor	Alternating cycles of learner-teacher feedback from positive to negative	Self-assessment<br>What was done well<br>Self-assessment what could be done better<br>Facilitator –alternate skills<br>Learner feedback to facilitator	POWER	Not addressed
**(Cantillon and Sargeant 2008)**	Critique of feedback sandwich and Pendleton Model	Reflective dialogue	Student perspective<br>Teacher perspective<br>Learner Reflection on improvement<br>Teacher elaboration /correction	POWE	Not addressed
**R2C2 (Sargeant et al. 2015, p. 2, 2017, p. 2)**	Supervision/<br>Original study involving program design with evaluation.	Coaching - facilitative model of informed self- assessment incorporating the science of behaviour change	R1- Building rapport and relationship; R2 - explore Reactions and perceptions; C1 - Explore physician understanding of the Content of the data report; C2 - Coach for performance change	E POWER	Not addressed
**(Nicol and Macfarlane-Dick 2006)**	Conceptual model not framework	Informed self-assessment	Clarify standard<br>Facilitate self-assessment<br>Deliver information<br>Dialogue	PO	Not addressed

			Motivation self esteem<br>Opportunities<br>Receive feedback on process		
**(Johnson et al. 2016)**	Literature review and consensus of good supervision behaviours	Not intended as a framework<br>Establishing an effective learning environment<br>identify performance gap, action planning	Setup<br>Assessment<br>Action plan	Prepare<br>MPO WE<br>Follow up	Non-judgemental, supportive, trustworthy, specific comments
**SET-GO – Silverman 1997 (Kurtz and Silverman 1996)**	Supervision / Multidisciplinary	Goal orientated with options consistent with coaching model	What I **s**aw<br>What **e**lse did you see<br>What do you **t**hink<br>What **g**oals<br>Any **o**ffers	POWE	Not addressed

Table1(c) Feedback Models

#The conversation goals addressed from the prepare-EMPOWER-enact model are represented by the appropriate letter. See manuscript for details Table 1.

The strategies proposed are listed with the abbreviations: Active listening (AL); Non-verbal strategies (NV); Observation-based Enquiry (OE); Advocacy Inquiry (AI). See manuscript for further details.

Model name/ Author	Context/ Target audience/ Publication type/ Description	key elements of framework	Structure	Conversational goals addressed #	Strategies proposed ##
**Comskil (Brown and Bylund 2008)**	Patient interview context but more broadly applicable. Proposes a framework to guide curriculum development based on Goal, planning action theory	Separates goals, strategies , skills, process tasks and cognitive appraisals	As per proposed framework with the addition of process tasks (create an environment for good communication) and cognitive appraisals , which include responding to patient cues and identifying and responding to barriers	Depends on conversation e.g. breaking bad news :" promotes understanding, recall, and a sense of ongoing support."	Strategies enlisted for specific goals
**COMFORT (Villagran et al. 2010)**	Breaking Bad news model based on interaction-adaption theory	COMFORT serves as the framework for BBN: communication, orientation, mindfulness, family, ongoing, reiterative messages, and team.	Not a linear guide but a set of competencies	Comfort, Verbal clarity , healing presence , anticipating anxieties	Mindfulness, involvement of family, re-iteration, involvement of team, follow up

Table 1(d) Publications identified during review process.

References

Ahmed, M., Arora, S., Russ, S., Darzi, A., Vincent, C., & Sevdalis, N. (2013). Operation Debrief: A SHARP Improvement in Performance Feedback in the Operating Room. *Annals of Surgery*, *258*(6), 958–963. https://doi.org/10.1097/SLA.0b013e31828c88fc

Arora, S., Ahmed, M., Paige, J., Nestel, D., Runnacles, J., Hull, L., et al. (2012). Objective structured assessment of debriefing: bringing science to the art of debriefing in surgery. *Annals of Surgery*, *256*(6), 982–988. https://doi.org/10.1097/SLA.0b013e3182610c91

Baile, W. F., Buckman, R., Lenzi, R., Glober, G., Beale, E. A., & Kudelka, A. P. (2000). SPIKES-A six-step protocol for delivering bad news: application to the patient with cancer. *The oncologist*, *5*(4), 302–311.

Brighton, L. J., Selman, L. E., Gough, N., Nadicksbernd, J., Bristowe, K., Millington-Sanders, C., & Koffman, J. (2018). 'Difficult Conversations': evaluation of multiprofessional training. *BMJ Supportive & Palliative Care*, *8*(1), 45–48. https://doi.org/10.1136/bmjspcare-2017-001447

Brown, R. F., & Bylund, C. L. (2008). Communication Skills Training: Describing a New Conceptual Model. *Academic Medicine*, *83*(1), 37. https://doi.org/10.1097/ACM.0b013e31815c631e

Cantillon, P., & Sargeant, J. (2008). Giving feedback in clinical settings. *Bmj*, *337*(nov10_2), a1961–a1961.

Cheng, A., Morse, K. J., Rudolph, J., Arab, A. A., Runnacles, J., & Eppich, W. (2016). Learner-Centered Debriefing for Health Care Simulation Education: Lessons for Faculty Development. *Simulation in Healthcare: The Journal of the Society for Simulation in Healthcare*, *11*(1), 32–40. https://doi.org/10.1097/SIH.0000000000000136

Chowdhury, R. R., & Kalu, G. (2004). Learning to give feedback in medical education. *The Obstetrician & Gynaecologist*, *6*(4), 243–247.

Clayton, J. M., Butow, P. N., Waters, A., Laidsaar-Powell, R. C., O'Brien, A., Boyle, F., et al. (2013). Evaluation of a novel individualised communication-skills training intervention to improve doctors' confidence and skills in end-of-life communication. *Palliative Medicine; London*, *27*(3), 236–43. http://dx.doi.org.libraryproxy.griffith.edu.au/10.1177/0269216312449683

Eppich, W., & Cheng, A. (2015). Promoting Excellence and Reflective Learning in Simulation (PEARLS): Development and Rationale for a Blended Approach to Health Care Simulation Debriefing. *Simulation in Healthcare: The Journal of the Society for Simulation in Healthcare*, *10*(2), 106–115. https://doi.org/10.1097/SIH.0000000000000072

Hesketh, E. A., & Laidlaw, J. M. (2002). Developing the teaching instinct, 1: Feedback. *Medical Teacher*, *24*(3), 245–248. https://doi.org/10.1080/014215902201409911

Husebø, S. E., O’Regan, S., & Nestel, D. (2015). Reflective Practice and Its Role in Simulation. *Clinical Simulation In Nursing*, *11*(8), 368–375. https://doi.org/10.1016/j.ecns.2015.04.005

Imperial College London. (2010). London Handbook for Debriefing: Imperial College. http://www1.imperial.ac.uk/resources/EE125DD5-63D9-48AB-8A77-F2951610CD83/lw2222ic_debrief_book_a5.pdf. Accessed 29 September 2017

Jaye, P., Thomas, L., & Reedy, G. (2015). “The Diamond”: a structure for simulation debrief. *The Clinical Teacher*, *12*(3), 171–175. https://doi.org/10.1111/tct.12300

Johnson, C. E., Keating, J. L., Boud, D. J., Dalton, M., Kiegaldie, D., Hay, M., et al. (2016). Identifying educator behaviours for high quality verbal feedback in health professions education: literature review and expert refinement. *BMC Medical Education*, *16*(1). https://doi.org/10.1186/s12909-016-0613-5

Keller, V. F., & Carroll, J. G. (1994). A new model for physician-patient communication. *Patient Education and Counseling*, *23*(2), 131–140.

Kurtz, S. M., & Silverman, J. D. (1996). The Calgary-Cambridge Referenced Observation Guides: an aid to defining the curriculum and organizing the teaching in communication training programmes. *Medical Education*, *30*(2), 83–89. https://doi.org/10.1111/j.1365-2923.1996.tb00724.x

Makoul, G. (2001). Essential Elements of Communication in Medical Encounters: The Kalamazoo Consensus Statement. *Academic Medicine*, *76*(4). http://journals.lww.com/academicmedicine/Fulltext/2001/04000/Essential_Elements_of_Communication_in_Medical.21.aspx

Milan, F. B., Parish, S. J., & Reichgott, M. J. (2006). A Model for Educational Feedback Based on Clinical Communication Skills Strategies: Beyond the “Feedback Sandwich.” *Teaching and Learning in Medicine*, *18*(1), 42–47. https://doi.org/10.1207/s15328015tlm1801_9

Nicol, D. J., & Macfarlane-Dick, D. (2006). Formative assessment and self-regulated learning: a model and seven principles of good feedback practice. *Studies in Higher Education*, *31*(2), 199–218. https://doi.org/10.1080/03075070600572090

Novack, D. H., Dubé, C., & Goldstein, M. G. (1992). Teaching Medical Interviewing: A Basic Course on Interviewing and the Physician-Patient Relationship. *Archives of Internal Medicine*, *152*(9), 1814–1820. https://doi.org/10.1001/archinte.1992.00400210046008

Phrampus, P. E., & O’Donnell, J. M. (2013). Debriefing Using a Structured and Supported Approach. In *The Comprehensive Textbook of Healthcare Simulation* (pp. 73–84). Springer, New York, NY. https://doi.org/10.1007/978-1-4614-5993-4_6

Rudland, J., Wilkinson, T., Wearn, A., Nicol, P., Tunny, T., Owen, C., & O’Keefe, M. (2013). A student-centred feedback model for educators. *The Clinical Teacher*, *10*(2), 99–102. https://doi.org/10.1111/j.1743-498X.2012.00634.x

Rudolph, J. W., Simon, R., Dufresne, R. L., & Raemer, D. B. (2006). There's no such thing as "nonjudgmental" debriefing: a theory and method for debriefing with good judgment. *Simulation in Healthcare: Journal of the Society for Simulation in Healthcare*, *1*(1), 49–55.

Sargeant, J., Lockyer, J., Mann, K., Holmboe, E., Silver, I., Armson, H., et al. (2015). Facilitated Reflective Performance Feedback: Developing an Evidence- and Theory-Based Model That Builds Relationship, Explores Reactions and Content, and Coaches for Performance Change (R2C2). *Academic Medicine: Journal of the Association of American Medical Colleges*. https://doi.org/10.1097/ACM.0000000000000809

Sargeant, J., Mann, K., Manos, S., Epstein, I., Warren, A., Shearer, C., & Boudreau, M. (2017). R2C2 in Action: Testing an Evidence-Based Model to Facilitate Feedback and Coaching in Residency. *Journal of Graduate Medical Education*, *9*(2), 165–170. https://doi.org/10.4300/JGME-D-16-00398.1

Sawyer, T. L., & Deering, S. (2013). Adaptation of the US Army's After-Action Review for simulation debriefing in healthcare. *Simulation in Healthcare: Journal of the Society for Simulation in Healthcare*, *8*(6), 388–397. https://doi.org/10.1097/SIH.0b013e31829ac85c

Smith, R. C., Marshall-Dorsey, A. A., Osborn, G. G., Shebroe, V., Lyles, J. S., Stoffelmayr, B. E., et al. (2000). Evidence-based guidelines for teaching patient-centered interviewing. *Patient Education and Counseling*, *39*(1), 27–36. https://doi.org/10.1016/S0738-3991(99)00088-9

Villagran, M., Goldsmith, J., Wittenberg-Lyles, E., & Baldwin, P. (2010). Creating COMFORT: A Communication-based model for Breaking Bad News. *Communication Education*, *59*(3), 220–234. https://doi.org/10.1080/03634521003624031

Waterson, L. (2013). Conversations in ClinicalSupervision Participant Manual Final May 16 2013 (2).pdf. Health Education Training Institute (NSW) Australia.

Zigmont, J. J., Kappus, L. J., & Sudikoff, S. N. (2011). The 3D model of debriefing: defusing, discovering, and deepening. *Seminars in Perinatology*, *35*(2), 52–58. https://doi.org/10.1053/j.semperi.2011.01.003
