# Supplementary material for: Finding common ground: meta-synthesis of communication frameworks found in patient communication, supervision and simulation literature
Source: BMC Med Educ. 2020 Feb 11;20:45. doi: 10.1186/s12909-019-1922-2 (PMC7014645; doi:10.1186/s12909-019-1922-2)
Supplement: Supplementary file 1 — Additional file 1. Evaluating the quality of communication frameworks. [file 12909_2019_1922_MOESM1_ESM.zip › Fining common ground BMC_MEDED Appendix R2R3.docx]

**APPENDIX 2:**

**Table 1**: Summaries of individual publications included in the review (a) Simulation models (b) Patient interview models (c) Feedback models and (d) identified in review

| Model name/ Author | Context/ Target audience/ Publication type/ Description | key elements of framework | Structure | Conversational goals addressed # | Strategies proposed ## |
| --- | --- | --- | --- | --- | --- |
| (Cheng et al. 2016) | Focus on learner centred approach | Focuses on Reactions and Analysis phase | Identify learner agenda  Prioritise content  Promote learner self- assessment  Explore rationale  Close performance gaps  Manage transitions/Manage time  Identify key messages | MPOWERS | AL, NVC, OE, |
| ALOBA –Silverman 1996 Reference (Chowdhury and Kalu 2004) | Supervision /  Multidisciplinary/ Critical review of existing models, described by authors as an agenda-led outcome-based analysis. | Rehearsal of skills to achieve outcomes;  Facilitator closes and summarises | Learner set goals;  Facilitator praises achievements and introduces theories;  Rehearses skills | POWES | Not addressed |
| Universal Student-Centred Interview Framework  (Waterson 2013) | Supervision and Simulation  Multidisciplinary/  Training manual/  Framework supports supervision conversations in a range of contexts including orientation, workplace assessment and feedback; following up critical incidents and performance management; delivering bad news to students. Delivered as a simulation course. | “Manage agenda proposed as a ‘preview’ of the conversation assuming supervisor/debriefer addresses pre-determined learning or other objectives. Preview outlines a structure for the conversation, includes a summary of key events/issues and sign posting what will be addressed | Preparation,  Open, Middle, Preview end, End | E,M,P,O,W,E,R,S | AL, NV, OE, AI |
| GAS (Phrampus and O’Donnell 2013) | Simulation / Multidisciplinary / Structured debriefing model; Gather, Analyze, Summarize in an operational acronym (GAS).  Highly structured with specific goals, actions and timeframes for each phase.  May use phased domain debriefing i.e. group teamwork then separated clinical debrief for each profession | Gather: learner reactions  Recap by team for shared mental model (25%)  Analyze: learner-centred reflection and analysis of actions. Facilitator observations and questions to identify performance gaps. (50%)  Summarize: learning points revisited / reviewed. (25%) | Gather  Analyze  Summarize | E,M,P,O,W,S | AL, NV, OE |
| 3D (Zigmont et al. 2011) | Simulation / Multidisciplinary / Structured Debriefing model  Diffusing, Discovering and Deepening.  Authors note relevance to supervision in addition to simulation | Feelings included in Diffuse (2^nd^ stage)  Ground rules; Format expectations;  Explore/Address emotions;  Needs analysis; Discovering phases uses reflective principles to elicit and share and shape mental frames;  Prompt individual to apply new knowledge to practice;  Summarise key learning points;  Recap | Prebrief ground rules Diffuse  Discovering  Deepening  Summarise | E,M,P,O,W,E,R,S | AL, NV, OE, AI |
| Diamond (Jaye et al. 2015) | Simulation / Multidisciplinary / Structured debriefing model with script: three phases presented in a diamond shape to indicate relative timeframes with clear transitions between each phase. | Feelings de-emphasised as culturally not required in UK; Clarify what good performance is; Facilitate self-assessment; Provide opportunities to close the gap; Include discussion of feedback with peers and supervisors; Elicit feedback on process to aid teaching; Use feedback to improve teaching | Description  Transition: clarification Analysis  Transition: reinforce agreements  Application | M,P,O,W,E,R,S | AL, NV, OE |
| PEARLS (Eppich and Cheng 2015) | Simulation / Multidisciplinary /  Structured debriefing model with script. Four phases: reactions, description, analysis, summary. | Integrated model considering context-specific factors influencing the choice of approach to analysis phase (more learner or more facilitator directed), including time available, whether learners’ rationale for action is clear, and whether the learning objective/performance gap are related to knowledge, skills, or behaviours. | Reactions  Description  Analysis  Summary / application | E,M,P,O,W,E,R,S | AL, NV, OE, AI |
| AAR (Sawyer and Deering 2013) | Simulation/ Multidisciplinary /  Structured debriefing model / Seven phase After Action Review (AAR) of performance based upon the US army debrief model. Uses acronym DEBRIEF | Explicit review of learning objectives and expected performance against a standard. Shared mental model of critical aspects of case  Identifying what and why reveals underlying mental model and facilitates change. Prompt to what would do differently next time | Define the rules  Explain learning objectives  Benchmark performance  Review expected actions  Identify what happened  Examine why  Formalise learning | M,P,O,W,E,R | AL, NV, OE, AI |
| SHARP (Ahmed et al. 2013) | Supervision / simulation / Surgical trainees (potential wider application)  Pocket tool to focus feedback on learning objectives established prior to case. Aims to make feedback specific and learner centred.  Designed as a brief reminder | Learner centred – objectives set by learner. Agenda is managed by following tool that both learner and supervisor have. Observations based upon what the learner set out to do and feedback specific to that. Plan for next time articulated. Feelings not included. Summary of learning not specified | **S**et learning objectives (before case) with learner  **H**ow did it go (after case) – compared to expected  **A**ddress concerns – what didn’t go well  **R**eview learning points – met objectives?  **P**lan ahead | M,P,O,W,R | AL, OE |
| OSAD (Arora et al. 2012; Imperial College London 2010) | Simulation / Multidisciplinary / debriefers  Objective Structured Assessment of Debriefing (OSAD) tool.  Used as a how to guide for novice debriefers, rating tool of feedback quality and research tool to identify if intervention to improve debriefing is effective | Rates debrief in areas of:  Approach / Learning Environment / Engagement / Reflection /Reaction / Analysis / Diagnosis / Application  Provides anchored rating scale with descriptors of poor, intermediate and good applications. Emphasis on learner centeredness, targeting behaviours that can be changed and application to practice. No diffusing of feelings prior to reflection. | Open: environment & approach  Middle: reflection, reaction, analysis and diagnosis  End: summary and application | Components of “good” rating scale includes  E,M,P,O,W,E,R,S | AL, NV, OE |
| Gibbs (Husebø et al. 2015) | Simulation /  Multidisciplinary / Review of Gibbs’ Reflective Cycle theory as applied to debriefing in simulation | Six stage reflective cycle with each stage informing the next. Provides script for each stage. Original model for self-evaluation and reflection so does not emphasise facilitator observations  Description / Feelings / Evaluation / Analysis / Conclusion / Action Plan | Open: case description and feelings about case  Middle: evaluation and analysis of performance, drawing conclusions  End: Action plan | E,M,P,W,E,R | AL |
| Debrief with Good Judgement (Rudolph et al. 2006) | Simulation / Multidisciplinary / Structured debriefing model / three phase model with an emphasis on debriefing stance. | Reactions phase for learners to “blow off steam” and provide insight to debriefer. Demonstrate respect for learners through using topics raised in reactions phase as part of debrief. Signpost topic changes. Analysis phase four step process of observe, feedback, investigate and close performance gap. Synthesises and augments improvement suggestions with evidence and experience. Codify insights into “rules of thumb” to improve practice. | Reactions  Analysis  Summary | E,M,P,O,W,E,R,S | AL, NV, OE, AI |

**Table 1 (a).** Simulation models

#The conversation goals addressed from the prepare-EMPOWER-enact model are represented by the appropriate letter. See manuscript for details Table 1.

## The strategies proposed are listed with the abbreviations: Active listening (AL); Non-verbal strategies (NV); Observation-based Enquiry (OE); Advocacy Inquiry (AI). See manuscript for further details.

| Model name/ Author | Context/ Target audience/ Publication type/ Description | key elements of framework | Structure | Conversational goals addressed # | Strategies proposed ## |
| --- | --- | --- | --- | --- | --- |
| -PREPARED (Clayton et al. 2013) | End of life discussions analysis of 22 workshop participants | Detailed observable descriptors of specific behaviours to facilitate assessment. Specific content requirements , less emphasis on structure, more on method | Prepares  Relates  Explores  Provides  Acknowledges Emotion and concerns  Encourages questions  Documents | EPO | Encourages questions  Anticipates and Addresses fears and concerns  Check understanding |
| Scars(Brighton et al. 2018) | End of life discussions analysis of 886 workshop participants | Focus on clarity and compassion framework elaborated with a workshop discussion | Setting, communicate with kindness, ask respond and reflect, summary and plan | Prepare  EMPORS | Not elaborated within manuscript |
| (Keller and Carroll 1994) E4 | Four tasks of clinical communication | Structure not emphasised focus on patient education | Engage  Empathise  Educate  Enlist | E M O W E | Open ended questions , non verbal , acknowledgement, self disclosure |
| (Kurtz and Silverman 1996) Calgary Cambridge | Commonly used framework for teaching patient interviewing | Content v process  Providing structure and  Building the relationship  Patients perspective and biomedical perspective  Achieving a shared understanding | Initiating session  Gathering information Physical examination  Explanation and planning Closing the session | Preparation  M P O W E R  Follow up | 70 core Process skills open to closed cone, active listening, facilitative response , cues, clarification , internal summary time-framing |
| Kalamazoo (Makoul 2001) | Consensus amalgamation of 5 commonly used patient communication models | 7 tasks. Each task associated with desired behaviours | Build a relationship  Open discussion  Gather information  Understand the patient’s perspective  Share information  Reach agreement on problems and plans  Provide closure | E POWRS –Follow up | AL, open-closed, encourage participation as desired, personal connection , identify resources |
| (Novack et al. 1992)(BIC) | Specific behaviours constitute an interview checklist of 32 skills or behaviours | Separate stages and types of skills. Types of skills: facilitation; relationship; patient education. | Stages: opening; Information gathering; closing. | EMPO W  Follow up | Support, respect, acknowledges emotions , NVC , summarise, open-closed |

| (Smith et al. 2000) Patient- centred Interview | Method evaluated in RCT | Divides into 5 stages. Describes tasks associated with each stage | Setting the stage  Chief complaint  Non focused interviewing  Focused interviewing  Transition to Doctor centred |  |  |
| --- | --- | --- | --- | --- | --- |
| SPIKES (Baile et al. 2000) | Breaking bad news |  | Setup  Perspective  Invitation  Knowledge  Empathy  Summary | Prepare  EMPORS | Assess information preferences, empathetic statements , exploratory statements, validation |

**Table 1(b)** Patient Interview models

#The conversation goals addressed from the prepare-EMPOWER-enact model are represented by the appropriate letter. See manuscript for details Table 1.

## The strategies proposed are listed with the abbreviations: Active listening (AL); Non-verbal strategies (NV); Observation-based Enquiry (OE); Advocacy Inquiry (AI). See manuscript for further details.

| Model name/ Author | Context/ Target audience/ Publication type/ Description | key elements of framework | Structure | Conversational goals addressed # | Strategies proposed ## |
| --- | --- | --- | --- | --- | --- |
| (Milan et al. 2006) | Feedback based on clinical communication (using “PEARLS” –not simulation model) | Change model with loose structure and coaching strategies | Loosely structured s | E | Partnership  Empathic understanding  Apology for barriers  Respect values and choices  Legitimisation  Support correction |
| (Hesketh and Laidlaw 2002) | Deal with formal and informal feedback | Mixture of stages and strategies | Prepare  Respect  Self-assess  Observations  Solutions  Check agreement | Prepare  PO | Non-judgemental language, respect, engagement, specific examples |
| (Rudland et al. 2013) | Student centred model not a conversation structure | Interaction between structural elements  Student characteristics of being responsive, receptive and reflective | Student characteristics  Contextual factors  Supervision characteristics and quality of feedback | W | Precise  Relevant  Outcome based  Measurable  Possible  Time determined  Encouraging  Descriptive |
| Pendletons rules 1984 referenced in (Chowdhury and Kalu 2004) | Feedback supervisor facilitator or supervisor | Alternating cycles of learner-teacher feedback from positive to negative | Self-assessment  What was done well  Self-assessment what could be done better  Facilitator –alternate skills  Learner feedback to facilitator | POWER | Not addressed |
| (Cantillon and Sargeant 2008) | Critique of feedback sandwich and Pendleton Model | Reflective dialogue | Student perspective  Teacher perspective  Learner Reflection on improvement  Teacher elaboration /correction | POWE | Not addressed |
| R2C2 (Sargeant et al. 2015, p. 2, 2017, p. 2) | Supervision/  Original study involving program design with evaluation. | Coaching - facilitative model of informed self- assessment incorporating the science of behaviour change | R1- Building rapport and relationship; R2 - explore Reactions and perceptions; C1 -Explore physician understanding of the Content of the data report; C2 - Coach for performance change | E POWER | Not addressed |
| (Nicol and Macfarlane‐Dick 2006) | Conceptual model not framework | Informed self-assessment | Clarify standard  Facilitate self- assessment  Deliver information  Dialogue  Motivation self esteem  Opportunities  Receive feedback on process | PO | Not addressed |
| (Johnson et al. 2016) | Literature review and consensus of good supervision behaviours | Not intended as a framework  Establishing an effective learning environment  identify performance gap,  action planning | Setup  Assessment  Action plan | Prepare  MPO WE  Follow up | Non-judgemental, supportive, trustworthy, specific comments |
| SET-GO – Silverman 1997 (Kurtz and Silverman 1996) | Supervision /  Multidisciplinary | Goal orientated with options consistent with coaching model | What I **s**aw  What **e**lse did you see  What do you **t**hink  What **g**oals  Any **o**ffers | POWE | Not addressed |

**Table1(c)** Feedback Models

#The conversation goals addressed from the prepare-EMPOWER-enact model are represented by the appropriate letter. See manuscript for details Table 1.

## The strategies proposed are listed with the abbreviations: Active listening (AL); Non-verbal strategies (NV); Observation-based Enquiry (OE); Advocacy Inquiry (AI). See manuscript for further details.

| Model name/ Author | Context/ Target audience/ Publication type/ Description | key elements of framework | Structure | Conversational goals addressed # | Strategies proposed ## |
| --- | --- | --- | --- | --- | --- |
| Comskil (Brown and Bylund 2008) | Patient interview context but more broadly applicable. Proposes a framework to guide curriculum development based on Goal, planning action theory | Separates goals, strategies , skills, process tasks and cognitive appraisals | As per proposed framework with the addition of process tasks (create an environment for good communication) and cognitive appraisals , which include responding to patient cues and identifying and responding to barriers | Depends on conversation e.g. breaking bad news :” promotes understanding, recall, and a sense of ongoing support.” | Strategies enlisted for specific goals |
| COMFORT (Villagran et al. 2010) | Breaking Bad news model based on interaction-adaption theory | COMFORT serves as the  framework for BBN: communication, orientation, mindfulness, family, ongoing,  reiterative messages, and team. | Not a linear guide but a set of competencies | Comfort, Verbal clarity , healing presence , anticipating anxieties | Mindfulness, involvement of family, re-iteration, involvement of team, follow up |

**Table 1(d)** Publications identified during review process.

## References

Ahmed, M., Arora, S., Russ, S., Darzi, A., Vincent, C., & Sevdalis, N. (2013). Operation Debrief: A SHARP Improvement in Performance Feedback in the Operating Room. *Annals of Surgery*, *258*(6), 958–963. https://doi.org/10.1097/SLA.0b013e31828c88fc

Arora, S., Ahmed, M., Paige, J., Nestel, D., Runnacles, J., Hull, L., et al. (2012). Objective structured assessment of debriefing: bringing science to the art of debriefing in surgery. *Annals of Surgery*, *256*(6), 982–988. https://doi.org/10.1097/SLA.0b013e3182610c91

Baile, W. F., Buckman, R., Lenzi, R., Glober, G., Beale, E. A., & Kudelka, A. P. (2000). SPIKES-A six-step protocol for delivering bad news: application to the patient with cancer. *The oncologist*, *5*(4), 302–311.

Brighton, L. J., Selman, L. E., Gough, N., Nadicksbernd, J., Bristowe, K., Millington-Sanders, C., & Koffman, J. (2018). ‘Difficult Conversations’: evaluation of multiprofessional training. *BMJ Supportive & Palliative Care*, *8*(1), 45–48. https://doi.org/10.1136/bmjspcare-2017-001447

Brown, R. F., & Bylund, C. L. (2008). Communication Skills Training: Describing a New Conceptual Model. *Academic Medicine*, *83*(1), 37. https://doi.org/10.1097/ACM.0b013e31815c631e

Cantillon, P., & Sargeant, J. (2008). Giving feedback in clinical settings. *Bmj*, *337*(nov10_2), a1961–a1961.

Cheng, A., Morse, K. J., Rudolph, J., Arab, A. A., Runnacles, J., & Eppich, W. (2016). Learner-Centered Debriefing for Health Care Simulation Education: Lessons for Faculty Development. *Simulation in Healthcare: The Journal of the Society for Simulation in Healthcare*, *11*(1), 32–40. https://doi.org/10.1097/SIH.0000000000000136

Chowdhury, R. R., & Kalu, G. (2004). Learning to give feedback in medical education. *The Obstetrician & Gynaecologist*, *6*(4), 243–247.

Clayton, J. M., Butow, P. N., Waters, A., Laidsaar-Powell, R. C., O’Brien, A., Boyle, F., et al. (2013). Evaluation of a novel individualised communication-skills training intervention to improve doctors’ confidence and skills in end-of-life communication. *Palliative Medicine; London*, *27*(3), 236–43. http://dx.doi.org.libraryproxy.griffith.edu.au/10.1177/0269216312449683

Eppich, W., & Cheng, A. (2015). Promoting Excellence and Reflective Learning in Simulation (PEARLS): Development and Rationale for a Blended Approach to Health Care Simulation Debriefing. *Simulation in Healthcare: The Journal of the Society for Simulation in Healthcare*, *10*(2), 106–115. https://doi.org/10.1097/SIH.0000000000000072

Hesketh, E. A., & Laidlaw, J. M. (2002). Developing the teaching instinct, 1: Feedback. *Medical Teacher*, *24*(3), 245–248. https://doi.org/10.1080/014215902201409911

Husebø, S. E., O’Regan, S., & Nestel, D. (2015). Reflective Practice and Its Role in Simulation. *Clinical Simulation In Nursing*, *11*(8), 368–375. https://doi.org/10.1016/j.ecns.2015.04.005

Imperial College London. (2010). London Handbook for Debriefing: Imperial College. http://www1.imperial.ac.uk/resources/EE125DD5-63D9-48AB-8A77-F2951610CD83/lw2222ic_debrief_book_a5.pdf. Accessed 29 September 2017

Jaye, P., Thomas, L., & Reedy, G. (2015). “The Diamond”: a structure for simulation debrief. *The Clinical Teacher*, *12*(3), 171–175. https://doi.org/10.1111/tct.12300

Johnson, C. E., Keating, J. L., Boud, D. J., Dalton, M., Kiegaldie, D., Hay, M., et al. (2016). Identifying educator behaviours for high quality verbal feedback in health professions education: literature review and expert refinement. *BMC Medical Education*, *16*(1). https://doi.org/10.1186/s12909-016-0613-5

Keller, V. F., & Carroll, J. G. (1994). A new model for physician-patient communication. *Patient Education and Counseling*, *23*(2), 131–140.

Kurtz, S. M., & Silverman, J. D. (1996). The Calgary-Cambridge Referenced Observation Guides: an aid to defining the curriculum and organizing the teaching in communication training programmes. *Medical Education*, *30*(2), 83–89. https://doi.org/10.1111/j.1365-2923.1996.tb00724.x

Makoul, G. (2001). Essential Elements of Communication in Medical Encounters: The Kalamazoo Consensus Statement. *Academic Medicine*, *76*(4). http://journals.lww.com/academicmedicine/Fulltext/2001/04000/Essential_Elements_of_Communication_in_Medical.21.aspx

Milan, F. B., Parish, S. J., & Reichgott, M. J. (2006). A Model for Educational Feedback Based on Clinical Communication Skills Strategies: Beyond the “Feedback Sandwich.” *Teaching and Learning in Medicine*, *18*(1), 42–47. https://doi.org/10.1207/s15328015tlm1801_9

Nicol, D. J., & Macfarlane‐Dick, D. (2006). Formative assessment and self‐regulated learning: a model and seven principles of good feedback practice. *Studies in Higher Education*, *31*(2), 199–218. https://doi.org/10.1080/03075070600572090

Novack, D. H., Dubé, C., & Goldstein, M. G. (1992). Teaching Medical Interviewing: A Basic Course on Interviewing and the Physician-Patient Relationship. *Archives of Internal Medicine*, *152*(9), 1814–1820. https://doi.org/10.1001/archinte.1992.00400210046008

Phrampus, P. E., & O’Donnell, J. M. (2013). Debriefing Using a Structured and Supported Approach. In *The Comprehensive Textbook of Healthcare Simulation* (pp. 73–84). Springer, New York, NY. https://doi.org/10.1007/978-1-4614-5993-4_6

Rudland, J., Wilkinson, T., Wearn, A., Nicol, P., Tunny, T., Owen, C., & O’Keefe, M. (2013). A student-centred feedback model for educators. *The Clinical Teacher*, *10*(2), 99–102. https://doi.org/10.1111/j.1743-498X.2012.00634.x

Rudolph, J. W., Simon, R., Dufresne, R. L., & Raemer, D. B. (2006). There’s no such thing as “nonjudgmental” debriefing: a theory and method for debriefing with good judgment. *Simulation in Healthcare: Journal of the Society for Simulation in Healthcare*, *1*(1), 49–55.

Sargeant, J., Lockyer, J., Mann, K., Holmboe, E., Silver, I., Armson, H., et al. (2015). Facilitated Reflective Performance Feedback: Developing an Evidence- and Theory-Based Model That Builds Relationship, Explores Reactions and Content, and Coaches for Performance Change (R2C2). *Academic Medicine: Journal of the Association of American Medical Colleges*. https://doi.org/10.1097/ACM.0000000000000809

Sargeant, J., Mann, K., Manos, S., Epstein, I., Warren, A., Shearer, C., & Boudreau, M. (2017). R2C2 in Action: Testing an Evidence-Based Model to Facilitate Feedback and Coaching in Residency. *Journal of Graduate Medical Education*, *9*(2), 165–170. https://doi.org/10.4300/JGME-D-16-00398.1

Sawyer, T. L., & Deering, S. (2013). Adaptation of the US Army’s After-Action Review for simulation debriefing in healthcare. *Simulation in Healthcare: Journal of the Society for Simulation in Healthcare*, *8*(6), 388–397. https://doi.org/10.1097/SIH.0b013e31829ac85c

Smith, R. C., Marshall-Dorsey, A. A., Osborn, G. G., Shebroe, V., Lyles, J. S., Stoffelmayr, B. E., et al. (2000). Evidence-based guidelines for teaching patient-centered interviewing. *Patient Education and Counseling*, *39*(1), 27–36. https://doi.org/10.1016/S0738-3991(99)00088-9

Villagran, M., Goldsmith, J., Wittenberg-Lyles, E., & Baldwin, P. (2010). Creating COMFORT: A Communication-based model for Breaking Bad News. *Communication Education*, *59*(3), 220–234. https://doi.org/10.1080/03634521003624031

Waterson, L. (2013). Conversations in ClinicalSupervision Participant Manual Final May 16 2013 (2).pdf. Health Education Training Institute (NSW) Australia.

Zigmont, J. J., Kappus, L. J., & Sudikoff, S. N. (2011). The 3D model of debriefing: defusing, discovering, and deepening. *Seminars in Perinatology*, *35*(2), 52–58. https://doi.org/10.1053/j.semperi.2011.01.003
